# Resistance Analysis of Low-Level Virologic Rebound During HIV-1 Treatment With Lenacapavir and Broadly Neutralizing Antibodies Teropavimab and Zinlirvimab

**DOI:** 10.1093/infdis/jiaf559

**Published:** 2025-11-05

**Authors:** Lisa Selzer, Sally Demirdjian, Brie Falkard, Jiani Li, Ross Martin, Sean E Collins, Joseph Eron, Laurie A VanderVeen, Christian Callebaut

**Affiliations:** Clinical Virology, Gilead Sciences, Inc., Foster City, California, USA; Clinical Virology, Gilead Sciences, Inc., Foster City, California, USA; Clinical Virology, Gilead Sciences, Inc., Foster City, California, USA; Bioinformatics, Gilead Sciences, Inc., Foster City, California, USA; Bioinformatics, Gilead Sciences, Inc., Foster City, California, USA; Clinical Development, Gilead Sciences, Inc., Foster City, California, USA; Division of Infectious Diseases, University of North Carolina, Chapel Hill, North Carolina, USA; Clinical Virology, Gilead Sciences, Inc., Foster City, California, USA; Clinical Virology, Gilead Sciences, Inc., Foster City, California, USA

**Keywords:** lenacapavir, teropavimab, zinlirvimab, HIV, resistance

## Abstract

**Background:**

High rates of virologic suppression were observed in the Phase 1b study (NCT04811040) investigating lenacapavir and two broadly neutralizing antibodies (bNAb), teropavimab (30 mg/kg) and zinlirvimab (10 or 30 mg/kg), in virologically suppressed people with HIV-1 susceptible (IC_90_ ≤ 2 μg/mL) to both (primary cohort, *n* = 20) or either (pilot cohort, *n* = 10) bNAb. We describe resistance analyses through Week (W) 26.

**Methods:**

Post-baseline resistance analyses were conducted at virologic failure, and exploratory resistance analyses performed for participants with virologic rebound. Low copy number genotyping methods for capsid and a 1 kb stretch of gp120 from rebound virus were developed, and phenotypic susceptibility assessed.

**Results:**

Virologic failure was observed in 1/30 participants. This primary cohort participant had HIV RNA 155 copies/mL at W16 and developed Q67H in capsid (lenacapavir fold-change 4.7), without resistance to bNAbs; the participant resuppressed on oral antiretrovirals. Two pilot cohort participants, experienced virologic rebound at W26 (55 and 72 copies/mL) and restarted oral antiretrovirals. In exploratory analyses, neither had emergent lenacapavir resistance or altered bNAb susceptibility.

**Conclusions:**

Lenacapavir, teropavimab, and zinlirvimab maintained a high rate of virologic suppression through W26, with rare emergent lenacapavir resistance and no bNAb resistance, supporting further Phase 2 evaluation.

## Teaser

Resistance analysis from a Phase 1b study of lenacapavir with broadly neutralizing antibodies teropavimab and zinlirvimab showed rare emergent lenacapavir resistance and no bNAb resistance during 26 weeks of treatment, supporting further evaluation of this twice-yearly regimen for HIV treatment.

Antiretroviral therapy (ART) remains a lifelong requirement for people with human immunodeficiency virus 1 (HIV-1) (PWH), for whom there are effective treatments available but no cure [1]. Adhering to daily oral ART can be challenging due to barriers including pill fatigue, forgetfulness, side effects, stigma, and mental health challenges [2, 3]. Long-acting therapies are being developed to address these challenges [4]. One such long-acting agent is lenacapavir (LEN), the first-in-class HIV capsid (CA) inhibitor targeting early and late stages of the viral life cycle [5]. LEN is administered by twice-yearly subcutaneous (SC) injections, exhibits potent antiviral activity, and has demonstrated clinical efficacy combined with oral ART [5–7]. LEN is approved, in combination with other antiretrovirals, for heavily treatment-experienced adults with multidrug-resistant HIV and for use as pre-exposure prophylaxis [8, 9].

Synchronous long-acting therapy for PWH requires partner agents with similar pharmacokinetic properties to LEN. Broadly neutralizing antibodies (bNAbs) targeting HIV envelope (ENV) neutralize a wide variety of HIV [10, 11]. Two bNAbs, 3BNC117 targeting the CD4 binding site and 10–1074 targeting the V3 glycan on the HIV ENV protein gp120, demonstrated a direct, rapid antiretroviral effect in single-agent, dose-escalation studies [12–14]. Teropavimab (TAB; formerly 3BNC117-LS or GS-5423) and zinlirvimab (ZAB; formerly 10–1074-LS or GS-2872) have been modified to extend their half-lives, allowing for twice-yearly intravenous dosing [15]. Over 90% of subtype B HIV viruses are highly susceptible to TAB or ZAB, while >50% are highly susceptible to both (90% inhibitory concentration [IC_90_] of ≤2 μg/mL by PhenoSense® monoclonal antibody (mAb) assay) [16].

LEN, TAB, and ZAB have been studied in a proof-of-concept Phase 1b study (NCT04811040) as the first prospective, complete, twice-yearly combination treatment for HIV. Participants were enrolled across a primary and a pilot cohort of participants with HIV highly susceptible to both or one of the bNAbs, respectively [17, 18]. The interventions were well tolerated, with SC LEN-related injection site reactions being the most reported adverse events [17, 18]. Virologic suppression (VS; HIV RNA <50 copies/mL) per US Food and Drug Administration Snapshot Algorithm was maintained through Week 26 in 26/30 participants; 3/30 participants had low-level virologic rebound (VR) (HIV RNA ≥50 to <1000 copies mL), and 1/30 participants had no data in the Week 26 window [17, 18].

Here, we describe the Phase 1b resistance analysis through Week 26 in participants treated with a single dose of LEN, TAB, and ZAB.

## METHODS

### Study Overview

This completed, randomized Phase 1b study (NCT04811040), conducted at 13 US sites, comprised a primary cohort of participants with HIV highly susceptible to both TAB and ZAB [17], and a pilot cohort of participants with HIV highly susceptible to either TAB or ZAB (Supplementary Figure 1) [18]. The methodology and primary outcome were communicated previously [17, 18].

### Participant Selection and Dosing

Eligible participants were aged 18–65 years with HIV virologically suppressed (HIV RNA <50 copies/mL) for ≥18 months, taking oral ART for ≥2 years, and with a nadir CD4 count of ≥350 cells/μL and a CD4 count of ≥500 cells/μL at study entry. Participants had proviral phenotypic susceptibility to TAB and/or ZAB, defined as IC_90_ ≤ 2 μg/mL for each antibody using the PhenoSense® mAb Assay (Monogram Biosciences, South San Francisco, CA, USA). Participants stopped oral ART and were randomly assigned (1:1) on Day 1 to receive a single dose of SC LEN 927 mg (plus LEN 600 mg oral loading on Days 1 and 2), intravenous TAB 30 mg/kg, and either intravenous ZAB 10 mg/kg or 30 mg/kg. The ZAB dose was masked for participants and investigators.

### Baseline Resistance Analyses

#### HIV Proviral CA and ENV Genotyping

**Figure 1.**
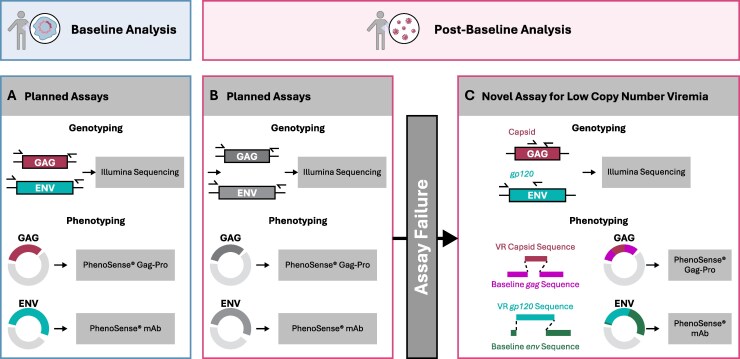
Summary of Genotyping and Phenotyping for Resistance Analyses. Planned genotypic and phenotypic analyses are presented for baseline (*A*) and post-baseline (*B*) analyses. In the event of assay failure for post-baseline planned genotypic and phenotypic analyses or for exploratory analyses, genotyping was conducted using a novel assay amenable to low virus copy number samples (*C*); to determine phenotypic susceptibility, the CA gene and the 1 kb stretch of gp120 sequenced from rebound viruses were synthesized and cloned into majority sequences for *gag* and *env* determined at baseline (*C*). Abbreviations: mAb, monoclonal antibody; VR, virologic rebound.

CA and ENV genotyping were performed at screening using the deep-sequencing HIV *gag* and *env* assays with variant detection limits of 2% and 1%, respectively (Seq-IT GmbH & Co. KG, Kaiserslautern, Germany) (Figure 1*A*). Briefly, DNA was extracted from purified peripheral blood mononuclear cells (PBMC), and then CA or ENV was amplified in bulk and sequenced using the Illumina MiSeq platform. Sequencing data were processed using a Gilead Sciences, Inc. developed analysis pipeline [19]. HIV-1 subtype was determined based on nucleotide identity of the consensus *env* gene against curated subtype reference sequences from GenBank® [20, 21].

#### HIV Proviral CA and ENV Phenotyping

Phenotypic HIV testing at screening was conducted on DNA extracted from PBMCs collected at screening (Figure 1*A*). HIV proviral CA was phenotyped with the PhenoSense® Gag-Pro assay (research use only; Monogram Biosciences) using the proviral consensus sequence of CA determined from deep sequencing. Baseline phenotypic susceptibility to TAB and ZAB was analyzed in real time by the PhenoSense® mAb DNA assay [22, 23], which evaluates the neutralization susceptibility of pseudovirions generated from PBMC-derived HIV ENV DNA amplified in bulk, to TAB or ZAB. The PhenoSense® mAb assay is Clinical Laboratory Improvement Amendments validated, and appropriate for clinical decision-making.

### Post-Baseline Resistance Analyses

Resistance analyses were performed on participants in the per-protocol defined resistance analysis population, which included participants who received at least 1 dose of the complete study drug regimen and met protocol-defined criteria for virologic failure (VF; HIV RNA ≥200 copies/mL on two consecutive visits, or HIV RNA ≥200 copies/mL at study discontinuation or Week 26). Exploratory resistance analyses were performed for participants meeting criteria for virologic rebound (VR; HIV RNA ≥50 copies/mL at any post-Day 1 visit, subsequently confirmed at the following scheduled or unscheduled visit, or HIV RNA ≥50 copies/mL at study discontinuation or Week 26) but did not meet requirements for per protocol resistance analysis population (Figure 1*B*, *C*).

#### HIV CA and ENV Genotyping From Plasma

Genotypic resistance testing of HIV CA and ENV was conducted at Monogram Biosciences using next-generation sequencing with reporting of variants present at >10% and >2% frequencies, respectively (GeneSeq® Gag-Pro and GenoSure® Env assays). In case of assay failure (AF), and for exploratory analyses, genotyping of *gag* and *env* was conducted for VF and VR participants using a novel genotyping assay developed at Gilead Sciences, Inc., amenable to low virus copy number samples (Figure 1*C*). For genotyping of HIV CA and ENV, RNA was isolated from 1.5 mL of plasma using the QIamp® Viral RNA Mini Kit (Qiagen, Hilden, Germany). Total RNA was divided into two parts and reverse transcribed with the SuperScript™ IV First-Strand Synthesis System (Invitrogen, Waltham, MA, USA) using gene-specific reverse primers for either CA or ENV (Supplementary Table 1). A partial 386 base pair (bp) stretch of the CA gene (HXB2 *gag* nucleotides 384–782; CA amino acids 1–128. GenBank: K03455.1) encompassing all known LEN resistance-associated residues, or the full CA gene (693 bp; HXB2 *gag* nucleotides 384–1193; CA amino acids 1–231. GenBank: K03455.1) were amplified via nested polymerase chain reaction using Platinum® Taq DNA High Fidelity Polymerase (Invitrogen, Waltham, MA, USA) (Supplementary Table 1). For all participants, a 994 bp stretch of ENV (HXB2 gp120 nucleotides 100–1093; ENV amino acids 34–371. GenBank: K03455.1) was amplified using the same method (Supplementary Table 1). HIV CA and ENV amplicons were deep sequenced using the Illumina platform (Novogene, Sacramento, CA, USA).

#### HIV CA and ENV Phenotyping

Phenotypic resistance testing was conducted at Monogram Biosciences using the PhenoSense® Gag-Pro and PhenoSense® mAb assays to assess responses to LEN and the bNAbs, respectively. In the event of AF, and for exploratory analyses, phenotyping of CA and ENV was conducted using a novel assay developed at Gilead Sciences, Inc. (Figure 1*C*). To determine phenotypic susceptibility, as described above, a partial capsid gene (386 bp) or the full capsid gene (693 bp) were sequenced from rebound viruses, synthesized, and cloned into majority sequences for *gag* determined at baseline. In addition, a 1 kb stretch of gp120 was sequenced from rebound viruses, synthesized, and cloned into majority sequences for *env* determined at baseline. Synthesis for both genes was based on the majority nucleotide call derived from deep sequencing. The chimeric *gag* and *env* genes were submitted to Monogram Biosciences for phenotyping using the PhenoSense® Gag-Pro and PhenoSense® mAb assays, respectively, with each gene assessed independently.

#### Genotypic Assessment of LEN and bNAb Susceptibility

For resistance analyses, CA resistance substitutions were defined according to the definitions of the International AIDS Society-USA (IAS-USA), as well as additional CA resistance substitutions reported in the literature [24–26]. For prediction of susceptibility to TAB and ZAB from genotypic data, baseline and post-baseline ENV sequences were analyzed for the presence of ENV amino acid signatures (Supplementary Table 2). Signatures were previously developed by combining neutralization data with virus sequence information to identify HIV ENV amino acid positions associated with increased susceptibility to TAB and ZAB [16]. This identification was performed by comparing the frequency of amino acids at each ENV residue in susceptible and non-susceptible viruses using genotypic-phenotypic datasets downloaded from the CATNAP database [27, 28] and internal Gilead datasets (susceptible, IC_50_ < 1 µg/mL; non-susceptible, IC_50_ ≥ 1 µg/mL) by Fisher's exact test [19].

#### Clonal Phenotypic Analysis at Baseline for Participant 1 With VF

Twenty-four single proviral *env* genes were amplified from the baseline screening sample and cloned into individual expression vectors. TAB or ZAB were titrated against the generated clonal pseudoviruses to determine concentrations required for virus inhibition [17].

#### Sensitivity to Additional bNAbs for Participant 1 With VF

Phenotypic resistance to five additional bNAbs for the baseline and post-baseline virus was assessed using the PhenoSense® mAb assay. The five additional bNAbs and their targets were N49P7 (CD4 binding site) [29], 1–18 (CD4 binding site) [30], PGT121 (HIV gp120 V3 base) [31], PG16 (V2/glycan apex region on the HIV ENV glycoprotein site) [32], and PGDM1400 (V2 apex region) [33].

## RESULTS

Details of the participant population have been reported previously [17, 18]. Baseline phenotypic data for HIV ENV confirmed eligibility of the 30 enrolled participants, and no participant had previous LEN exposure. All participants with available data (24/30 participants) had subtype B virus, except for one participant in the pilot cohort (30 mg/kg ZAB group), with subtype AG virus. During the study, 1/30 (3.3%) participants met criteria for VF and was included in the per-protocol resistance analysis population. Resistance testing was also performed for 2/30 (6.7%) participants who did not meet the criteria for resistance testing, but who experienced VR at Week 26 with HIV RNA <200 copies/mL.

### LEN, TAB, and ZAB Resistance Analyses

**Figure 2.**
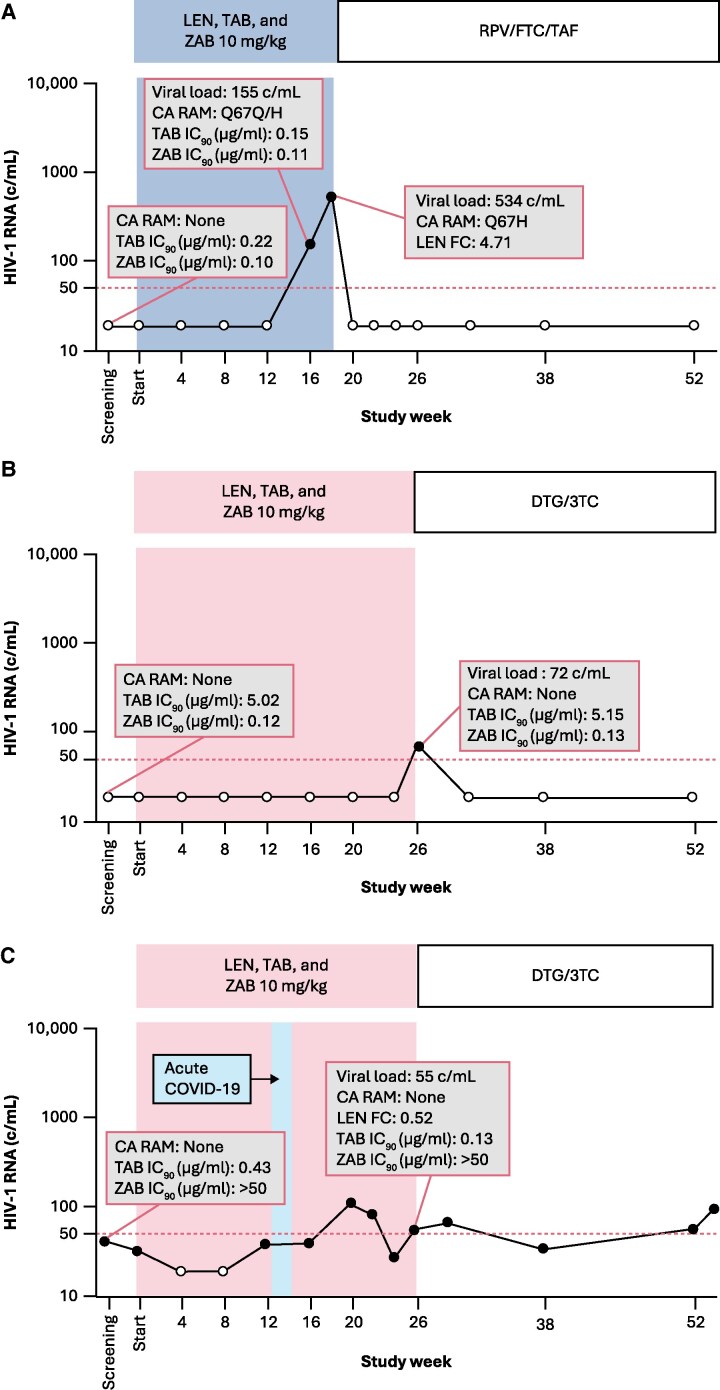
Viral Response and Resistance in the Three Participants With Virologic Rebound: (*A*) Participant 1, (*B*) Participant 2, and (*C*) Participant 3 viral load and resistance data, including genotypic and phenotypic data for CA and ENV, for the three participants with virologic rebound are presented. (*A*), Participant 1 from the Primary Cohort, (*B*) Participant 2, and (*C*) Participant 3 from the Pilot Cohort. *A*, For Participant 1, Q67H was detected as a mixed population at Week 16, with 84% H and 16% Q, and as 100% Q67H at the Week 16 retest visit (Week 16 + 15 d), based on deep sequencing data. Participants 1 and 2 resumed the same antiretroviral regimen that was in place before study entry, RPV/FTC/TAF and DTG/3TC, respectively. Participant 3 entered the study on ABC/DTG/3TC and resumed with DTG/3TC. Dotted line denotes the 50 copies/mL limit which defined virologic rebound in this study. Abbreviations: ABC/DTG/3TC, abacavir/dolutegravir/lamivudine; CA, capsid; COVID-19, Coronavirus-2019; DTG/3TC, dolutegravir/lamivudine; ENV, envelope; FC, fold change; IC_90_, 90% inhibitory concentration, LEN, lenacapavir; RAM, resistance associated mutation; RPV/FTC/TAF, rilpivirine/emtricitabine/tenofovir alafenamide; TAB, teropavimab; ZAB, zinlirvimab.

Descriptions of LEN, TAB, and ZAB resistance outcomes for the three participants are summarized in Table 1, with timing of viral load changes and resistance shown in Figure 2. The participant included in the per-protocol resistance analysis population (Participant 1) was from the primary cohort 10 mg/kg ZAB group and had VF confirmed with HIV RNA 534 copies/mL at the Week 16 retest visit (Week 16 + 15 days). Planned resistance testing methods resulted in AF likely due to low plasma viral load; therefore, resistance was evaluated by novel techniques using manual extraction of plasma samples and sequencing and synthesis of the CA and ENV genes. The participant, for whom a partial CA gene was analyzed (386 bp stretch of the capsid gene encompassing all known LEN resistance-associated residues), developed genotypic and phenotypic resistance to LEN; Q67H mixture with wild-type was detected at Week 16, which converted to a full mutation at the retest visit, with a LEN fold change of 4.71 (Figure 2*A*). No genotypic or phenotypic resistance to TAB or ZAB was observed. Participant 1 restarted oral ART at Week 18 (rilpivirine/emtricitabine/tenofovir alafenamide) and resuppressed by Week 20 (HIV RNA ≤50 copies/mL), with suppression maintained through subsequent study visits.

**Table 1.** Summary of Participants Included in the Resistance Analysis

Participant	Cohort	HIV Subtype	Treatment Group	Baseline			Post-baseline					
				TAB IC_90_ (μg/mL)	ZAB IC_90_ (μg/mL)	LEN in-vitro FC (Gag-Pro)^a^	Virologic rebound visit	HIV RNA at virologic rebound visit (copies/mL)	Emergent LEN RAMs	LEN in-vitro FC (Gag-Pro)^a,b^	In-vitro FC from baseline (PhenoSense® mAb)^b^	
											TAB	ZAB
1	Primary	B	LEN + TAB + ZAB 10 mg/kg	0.22	0.10	0.75	Week 16	155	Q67Q/H	ND	0.66	1.18
							Week 16 retest	534	Q67H	4.71	ND^c^	ND^c^
2	Pilot	B	LEN + TAB + ZAB 10 mg/kg	5.02^d^	0.12	AF	Week 26	72	None	AF	1.02	1.09
3	Pilot	B	LEN + TAB + ZAB 10 mg/kg	0.43	>50^e^	0.67	Week 26	55	None	0.52	0.30	1.00

^a^Fold change relative to wild-type HIV strain NL4.3.

^b^Phenotyping of *gag* and *env* was performed at virologic rebound based on the majority sequence determined from genotyping.

^c^PhenoSense mAb could not be determined due to assay failure during genotyping.

^d^Reduced susceptibility to TAB.

^e^Full resistance to ZAB.

Abbreviations: AF, assay failure; FC, fold change, IC_90_, 90% inhibitory concentration, LEN, lenacapavir; mAb, monoclonal antibody; ND, not determined; RAM, resistance associated mutation; TAB, teropavimab; ZAB, zinlirvimab.

The other two participants (Participants 2 and 3) were from the pilot cohort 10 mg/kg ZAB group [18]. Participant 2 was highly susceptible to ZAB (IC_90_ 0.12 μg/mL) but not TAB (IC_90_ 5.02 μg/mL) at baseline and had VR at Week 26. Participant 3 was highly susceptible to TAB (IC_90_ 0.43 μg/mL) but not ZAB (IC_90_ > 50 μg/mL) at baseline and experienced VR following acute COVID-19 during Weeks 12–14. Similarly to Participant 1, planned resistance testing could not be performed for Participants 2 and 3 on samples with low copy HIV RNA of 72 and 55 copies/mL, respectively. Novel resistance testing methods detected no treatment emergent resistance to LEN after analysis of the full CA gene, and no resistance to TAB or ZAB for either participant (Figure 2*B* and *C*). Per protocol, Participant 2 resumed oral ART at Week 26 (dolutegravir/lamivudine) and was suppressed at subsequent study visits (Figure 2*B*). Participant 3 resumed oral ART (dolutegravir/lamivudine) at Week 26 but continued to experience low-level viremia through Week 54 (Figure 2*C*).

ENV genotypic data for the three participants were evaluated to predict TAB and ZAB susceptibility based on the prevalence of ENV susceptibility signatures. Supplementary Table 3 provides a summary of the prevalence of baseline and post-baseline ENV susceptibility signatures, while a detailed amino acid alignment between baseline and post-baseline ENV sequences is shown in Supplementary Figure 2 and Supplementary Table 4. Minor differences between baseline and post-baseline genotypic signatures were observed for all three participants (Supplementary Table 3). For example, for Participant 2, who was only susceptible to ZAB, residue D325 was present at baseline but not Week 26. Similarly, for Participant 3, who was only susceptible to TAB, residue Y318 was present at baseline but not Week 26. Despite these changes in the susceptibility signatures, phenotypic susceptibility to TAB and ZAB were unaffected for Participant 2 and 3.

### Participant 1: Clonal Genotypic and Phenotypic Analyses

**Figure 3.**
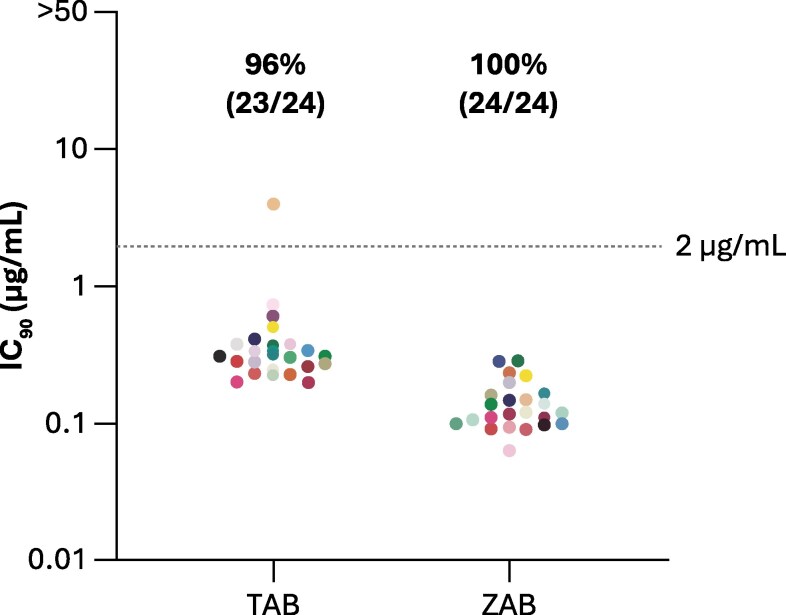
Clonal Phenotypic Baseline Resistance to TAB and ZAB in Participant 1 Virus. Clonal phenotyping (IC_90_) results illustrating TAB and ZAB susceptibility (IC_90_ ≤ 2 µg/mL) of 24 individually cloned, single proviral *env* genes from Participant 1's baseline screening sample. Dotted line represents the IC_90_ susceptibility cutoff of 2 μg/mL. IC_90_, 90% inhibitory concentration; TAB, teropavimab; ZAB, zinlirvimab.

For Participant 1, resistance analyses at the VF visit showed development of resistance to LEN (Q67H) but no change in susceptibility to either bNAb by phenotypic analysis of bulk amplified ENV. To further investigate potential pre-existing resistance to TAB and ZAB at baseline, phenotypic susceptibility to bNAbs was reassessed for clonal ENV sequences isolated from the screening visit sample. No phenotypic resistance to either bNAb was detected at baseline. Of 24 screened ENV clones, 23 had TAB IC_90_ ≤ 2 μg/mL and one (clone 17) had TAB IC_90_ of 3.96 μg/mL; all clones had ZAB IC_90_ ≤ 2 μg/mL. (Figure 3). For TAB, the bulk screening IC_90_ and clonal analysis geometric mean IC_90_ were 0.22 μg/mL and 0.36 (range, 0.20–3.96) μg/mL, respectively. For ZAB, the bulk screening IC_90_ and clonal analysis geometric mean IC_90_ were 0.26 μg/mL and 0.13 (range, 0.06–0.29) μg/mL, respectively.

Clonal sequence analysis showed that most TAB and ZAB signature residues were present in all clones, except for A281V in clone 7 and D325N in clone 22 (Supplementary Table 5). Phylogenetic analysis included full-length and 994 bp ENV regions for clonal sequences, along with Week 16 and Week 16 retest ENV sequences from Participant 1 (Supplementary Figure 3). The Week 16 ENV sequence was identical to that of clone 18 (TAB IC_90_:0.72 μg/mL, ZAB IC_90_:0.097 μg/mL), while the Week 16 retest sequence was closely related.

### Participant 1: Susceptibility of Provirus and Rebound Virus to Additional bNAbs

**Figure 4.**
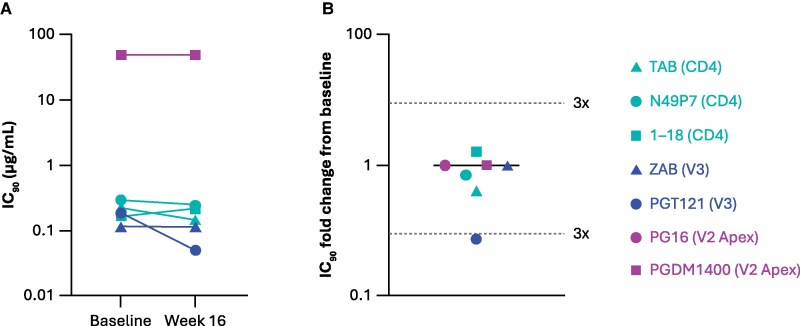
Minor Changes in Susceptibility to a Panel of bNAbs in Participant 1 Virus. (*A*), Phenotypic susceptibility of Participant 1's proviral DNA (baseline) and viral RNA at rebound (Week 16) to a panel of bNAbs. (*B*), Fold changes from baseline of phenotypic susceptibilities determined at Week 16. Abbreviations: bNAb, broadly neutralizing antibodies; IC_90_, 90% inhibitory concentration; TAB, teropavimab; ZAB, zinlirvimab.

To further investigate changes in virus susceptibility between baseline and the time of VF for Participant 1, provirus and rebound virus were phenotyped using five additional bNAbs targeting the CD4-binding site or the V3 or V2 Apex regions of ENV. Only minor changes in susceptibility between baseline and Week 16 were observed for bNAbs N49P7 (CD4), 1–18 (CD4), PGT121 (V3), PG16 (V2 Apex), and PGDM1400 (V2 Apex). IC_90_ values for most bNAbs showed <3-fold change from baseline (Figure 4); this is consistent with the minimal changes in susceptibility observed for TAB and ZAB between baseline and Week 16.

## DISCUSSION

In this Phase 1b study of LEN, TAB, and ZAB as the first twice-yearly HIV treatment, high rates of VS were maintained through 26 weeks, including among pilot cohort participants who were highly susceptible to only one bNAb [17, 18]. Emergent resistance was rare; of the three participants with VF or VR, all of whom received the lower dose of ZAB (10 mg/kg), treatment-emergent LEN resistance (Q67H) was only detected in one participant, with none showing treatment-emergent resistance to TAB or ZAB. The development of a novel low copy number genotyping assay allowed for genotypic and phenotypic resistance analyses in these three participants with low-level viremia (HIV RNA ≥50 to <1000 copies/mL), for whom attempts to sequence *gag* or *env* using planned assays resulted in AF or could not be performed due to low viral loads.

Participant 1 (primary cohort, low-dose ZAB group) developed the Q67H resistance-associated mutation (RAM) to LEN despite remaining susceptible to both bNAbs and having no pre-existing resistance mutations to LEN at baseline. Although the Q67H mutation has been observed in previous LEN treatment studies [6, 34–36], it is rarely seen without LEN exposure [37]. Naturally occurring LEN binding site variants in CA remain fully susceptible to LEN, indicating minimal impact of natural viral diversity on LEN efficacy [38]. LEN RAMs were identified in 19/258 PWH across three other clinical studies, including M66I, Q67H/K/N, K70H/N/R/S, N74D/H/K, A105S/T, and T107A/C/N/S [26, 34, 39]. Resistance typically emerged in highly treatment-experienced participants due to functional monotherapy from insufficient active antiretrovirals (ARVs) in, or inadequate adherence to, the optimized background regimen [5, 26, 35].

Resistance to the parent antibodies of TAB (3BNC117) and ZAB (10–1074) has been observed in the setting of monotherapy [12–14, 40, 41]. For 3BNC117, mutations in R456 and K282 were common in rebound viruses in participants receiving monotherapy [14], while resistance to 10–1074, was associated with mutations in the V3 loop binding site, ie, the ^324^GDIR^327^ motif and residues H330, N332, and S334 [13, 40]. Bioinformatic analyses of phenotypic neutralization and genotypic ENV data have identified ENV signatures important for viral neutralization by 3BNC117 and 10–1074 [19, 42]. For Participant 1, no development of phenotypic resistance or changes in these previously characterized resistance regions were detected. Clonal analysis of proviral ENV genes at baseline revealed no pre-existing geno- or phenotypic resistance to TAB or ZAB, consistent with the bulk ENV phenotyping performed at baseline. In addition, phylogenetic analysis revealed a close relationship between clone 18 (TAB and ZAB IC_90_ < 2 μg/mL) and the Week 16 and Week 16 Retest ENV sequences, indicating clone 18 may have emerged during viral rebound.

The emergence of resistance in Participant 1 is unlikely due to insufficient exposure to LEN, TAB, or ZAB, as the pharmacokinetic data and anti-drug antibodies were consistent with those of other study participants [17] and showed no impact on TAB or ZAB exposure or neutralization [17]. However, tissue distribution of LEN, TAB, and ZAB may have influenced viral rebound dynamics in this participant. Rebound could occur due to reactivation of latent viruses within tissue compartments, referred to as “sanctuary sites”; these sites may exhibit reduced penetration of ARVs, a highly debated topic in the field of HIV research [43, 44]. Interestingly, in clinical studies of cabotegravir plus the CD4-binding bNAb VRC07-523LS for maintenance in PWH, viral rebound in the absence of VRC07-523LS resistance was observed [45], suggesting that the distribution of long-acting ARVs in tissue compartments warrants further investigation.

No LEN resistance was detected in Participants 2 and 3 from the pilot cohort low-dose ZAB group. Similarly to Participant 1, no emergent phenotypic resistance to bNAbs or changes in known resistance-associated amino acid regions were observed. The unchanged phenotypic susceptibility to TAB and ZAB for Participants 2 and 3, despite observed changes in ENV susceptibility signatures, may be due to the signatures prioritizing specificity over sensitivity, which can lead to false negatives. Since LEN, TAB, and ZAB exposures for both participants were consistent with other study participants, the detection of low-level viremia (HIV RNA copies ≥50 copies/mL) in Participants 2 and 3 may be associated with a transient increase in detectable viral RNA levels due to viral activation or clonal expansion of the latent viral reservoir, rather than onward viral replication with development of resistance to the study drugs. This is particularly plausible for the participant who contracted COVID-19, which has been associated with episodes of low-level viremia in PWH [46].

These results need to be considered in the context of several study limitations. Phenotypic analyses at the time of viral rebound focused on the predominant viral variants. However, since viral diversity is typically low during initial rebound, these predominant variants likely provide a reliable representation of the overall viral population at this stage. For Participant 1, a portion of CA amino acids 1–128 was sequenced, although this includes all known LEN-associated resistance mutations. Additionally, only part of the gp120 ENV gene was amplified and sequenced, excluding the CD4 binding site; however, all previously determined susceptibility signature regions for 3BNC117 and 10–1074 were covered by the current sequence analysis [19]. Similarly, the V2 Apex region of ENV associated with binding to bNAbs PG16 and PGDM1400, tested for Participant 1, were covered by this analysis.

Overall, this Phase 1b study showed high rates of VS through 26 weeks of treatment with the triple therapy regimen, with low-level VR in three participants, all of whom were on the lower ZAB dose. Thus, LEN, TAB, and ZAB hold promise as a complete twice-yearly combination regimen for PWH. Evaluation of the investigational regimen with the higher ZAB dose is ongoing in a Phase 2 study in participants highly susceptible to both bNAbs (NCT05729568). Ongoing and future studies will evaluate how well baseline bNAb sensitivity translates into clinical outcomes.

## Supplementary Material

jiaf559_Supplementary_Data

## Supplementary Data

Supplementary materials are available at *The Journal of Infectious Diseases* online (http://jid.oxfordjournals.org/). Supplementary materials consist of data provided by the author that are published to benefit the reader. The posted materials are not copyedited. The contents of all supplementary data are the sole responsibility of the authors. Questions or messages regarding errors should be addressed to the author.

## Notes

***Acknowledgments.*** We extend our thanks to the study participants, their families, and the participating investigators and staff. Medical writing and editorial support were provided by Jessica Woods and Sherriden Beard of Ashfield MedComms (Macclesfield, UK), an Inizio company, and funded by Gilead Sciences, Inc.

***Data availability statement***. Gilead Sciences shares anonymized individual patient data upon request or as required by law or regulation with qualified external researchers based on submitted curriculum vitae and reflecting non-conflict of interest. The request proposal must also include a statistician. Approval of such requests is at Gilead Science's discretion and is dependent on the nature of the request, the merit of the research proposed, the availability of the data, and the intended use of the data. Data requests should be sent to datarequest@gilead.com.

***Financial support.*** This study was funded by Gilead Sciences, Inc., Foster City, CA, USA.
