# Supplementary material for: Resistance Analysis of Low-Level Virologic Rebound During HIV-1 Treatment With Lenacapavir and Broadly Neutralizing Antibodies Teropavimab and Zinlirvimab
Source: J Infect Dis. 2025 Nov 5;233(1):e212–20. doi: 10.1093/infdis/jiaf559 (PMC12811890; doi:10.1093/infdis/jiaf559)
Supplement: jiaf559_Supplementary_Data [file jiaf559_supplementary_data.docx]

Supplementary Materials

**Resistance Analysis of Low-Level Virologic Rebound During HIV-1 Treatment with Lenacapavir and Broadly Neutralizing Antibodies Teropavimab and Zinlirvimab**

Lisa Selzer, Sally Demirdjian, Brie Falkard, Jiani Li, Ross Martin, Sean E. Collins, Joseph Eron, Laurie A. VanderVeen, Christian Callebaut

**Supplementary Table 1. Primers used for nested PCR of *gag* and *env***

| Participant | Primer description | Primer sequence |
| --- | --- | --- |
| 1 | Fwd Gag outer | 5’-CAGTAGCAACCCTCTATTGTGTAC-3’ |
| 2, 3 | Fwd Gag outer | 5’-CAGTAGCAACCCTCTATTGTGTGC-3’ |
| 1 | Rev Gag outer | 5’-GTTCTATAGAATCGGTCTACATAGTCTC-3’ |
| 2 | Rev Gag outer* | 5’-CCTAGGGGCCCTGCAATGT-3’ |
| 3 | Rev Gag outer*# | 5’-CCTAGGGGCCCTGCAATTT-3’ |
| 1 | Fwd Gag inner | 5’-CAGCCAAAATTACCCTATAGTACAG-3’ |
| 2 | Fwd Gag inner | 5’-CAGCCAAAATTACCCTATAGTTCAG-3’ |
| 3 | Fwd Gag inner | 5’-CAGCCAAAATTACCCTATAGTGCAA-3’ |
| 1 | Rev Gag inner | 5’-ATTTCTCCTACTGGGATAGGTGG-3’ |
| 2 | Rev Gag inner | 5’-GTCTGCCACAGTTGAAACACTT-3’ |
| 3 | Rev Gag inner | 5’-CTTTGCCACAATTGAAACACTT-3’ |
| 1, 2, 3 | Fwd Env outer | 5’-TAGAAAGAGCAGAAGACAGTGGCAATGA-3’ |
| 1, 2 | Rev Env outer* | 5’-ACAGTAGAAAAATTCCCCTCCACAATTAA-3’ |
| 3 | Rev Env outer* | 5’-ACAGTAGAAAAATTCTCCTCCACAATTAA-3’ |
| 1 | Fwd Env inner | 5’-TTGTGGGTCACAGTCTATTATGGGGT-3’ |
| 2 | Fwd Env inner | 5’-TTGTGGGTCACAGTCCATTATGGAGT-3’ |
| 3 | Fwd Env inner | 5’-TTGTGGGTCACAGTTTATTATGGGGT-3’ |
| 1, 2, 3 | Rev Env inner | 5’-CAATTTCTGGGTCCCCTCCTGAGG-3’ |

Primers with * were used for cDNA reactions for the respective participants.

Primer with # was used for cDNA synthesis for Participant 1, but not for nested PCR.

Fwd, forward; PCR, polymerase chain reaction; Rev, reverse.

**Supplementary Table 2. ENV Susceptibility Signatures for TAB and ZAB**

| **ENV amino acid position** | **Positive predictive value^a^** |
| --- | --- |
| **TAB** | |
| No signature applied | 75 |
| I201 | 78 |
| I201/F353 | 84 |
| I108/I201/F353 | 86 |
| I108/I201/A281/F353 | 91 |
| E102/I108/I201/A281/F353 | 92 |
| E102/I108/I201/A281/Y318/F353 | 93 |
| **ZAB** | |
| No signature applied | 62 |
| N332 | 75 |
| N332/D325 | 80 |
| N332/D325/H330 | 83 |
| N332/D325/H330/T63 | 91 |
| N332/D325/H330/T63/T320 | 93 |
| N332/D325/H330/T63/T320/L179 | 97 |

^a^Predictive for IC_50_ <1 µg/mL (Moldt B, Parvangada A, Martin R, et al. Evaluation of Broadly Neutralizing Antibody Sensitivity by Genotyping and Phenotyping for Qualifying Participants to HIV Clinical Trials. *J Acquir Immune Defic Syndr*. 2021;88:61–69)
ENV, envelope; IC_50_, 50% inhibitory concentration; TAB, teropavimab; ZAB, zinlirvimab.

**Supplementary Table 3:** **Summary of bNAb Susceptibility Signatures**

|  | | **Env susceptibility signature present at frequency >99%** | | | | | | | | | | | |
| --- | --- | --- | --- | --- | --- | --- | --- | --- | --- | --- | --- | --- | --- |
|  |  | **TAB** | | | | | | **ZAB** | | | | | |
| Participant | Visit | I201 | I201 F353 | I108 I201 F353 | I108 I201 A281 F353 | E102 I108 I201 A281 F353 | E102 I108 I201 A281 Y318 F353 | N332 | N332  D325 | N332 D325 H330 | N332 D325 H330 T63 | N332 D325 H330 T63 T320 | N332 D325 H330 T63 T320 L179 |
| 1 | Baseline | No^a^ | No | No | No | No | No | Yes | Yes | Yes | Yes | No^b^ | No |
|  | Week 16 | Yes | Yes | Yes | Yes | Yes | Yes | Yes | Yes | Yes | Yes | Yes | Yes |
|  | Week 16 retest | Yes | Yes | Yes | Yes | Yes | Yes | Yes | Yes | Yes | Yes | Yes | Yes |
| 2 | Baseline | Yes | Yes | No | No | No | No | Yes | Yes | Yes | No | No | No |
|  | Week 26 | No^c^ | No | No | No | No | No | Yes | No | No | No | No | No |
| 3 | Baseline | Yes | Yes | Yes | Yes | Yes | Yes | Yes | No | No | No | No | No |
|  | Week 26 | Yes | Yes | Yes | Yes | Yes | No | Yes | No | No | No | No | No |

^a^I201 detected at 96.8%.

^b^T320 detected at 71.2%.

^c^I201 detected at 93%.
bNAb, broadly neutralizing antibody; TAB, teropavimab; ZAB, zinlirvirmab.

**Supplementary Table 4:** **Amino Acid Mixtures for Each Ambiguous Position**

| Participant | Visit | Position | Amino Acid Mixture |
| --- | --- | --- | --- |
| 1 | BL | 16 | R/K |
| 1 | BL | 133 | D/N |
| 1 | BL | 138 | S/N |
| 1 | BL | 141 | T/I |
| 1 | BL | 146 | T/A |
| 1 | BL | 148 | E/N/G/S/R/K/D |
| 1 | BL | 149 | M/I |
| 1 | BL | 177 | Q/P |
| 1 | BL | 182 | D/N |
| 1 | BL | 185 | R/I/S |
| 1 | BL | 214 | T/A |
| 1 | BL | 249 | I/V |
| 1 | BL | 300 | R/K |
| 1 | BL | 303 | H/R |
| 1 | BL | 308 | R/K |
| 1 | BL | 310 | I/F |
| 1 | BL | 313 | T/A |
| 1 | BL | 314 | G/E/D |
| 1 | BL | 315 | K/E/D/N |
| 1 | BL | 334 | K/N |
| 1 | BL | 338 | W/R |
| 1 | BL | 340 | F/V |
| 1 | BL | 344 | Q/R |
| 1 | BL | 355 | Q/R/K |
| 1 | BL | 365 | I/M/K |
| 1 | BL | 378 | D/N |
| 1 | BL | 379 | T/S |
| 1 | BL | 389 | K/E/D/N |
| 1 | BL | 390 | T/N |
| 1 | BL | 397 | R/S |
| 1 | BL | 400 | H/N |
| 1 | BL | 406 | R/K |
| 1 | BL | 509 | L/F |
| 1 | BL | 522 | L/V |
| 1 | BL | 531 | L/V |
| 1 | BL | 542 | L/M |
| 1 | BL | 573 | Y/F |
| 1 | BL | 599 | T/N |
| 1 | BL | 645 | Q/R |
| 1 | BL | 658 | S/N |
| 1 | BL | 661 | S/N |
| 1 | BL | 664 | K/N |
| 1 | BL | 704 | L/F |
| 1 | BL | 708 | L/F |
| 1 | BL | 733 | T/A |
| 1 | BL | 741 | T/A |
| 1 | BL | 748 | S/N |
| 1 | BL | 756 | H/R |
| 1 | Week 16 | 338 | W/* |
| 1 | Week 16 Retest | 51 | T/P |
| 1 | Week 16 Retest | 56 | S/A |
| 2 | BL | 30 | Q/K |
| 2 | BL | 229 | L/M |
| 2 | BL | 296 | S/N |
| 2 | BL | 355 | G/V |
| 2 | BL | 443 | R/L |
| 2 | BL | 458 | Y/C |
| 2 | BL | 499 | Y/* |
| 2 | BL | 724 | Q/H |
| 2 | BL | 773 | F/S |
| 2 | BL | 779 | A/T |
| 3 | BL | 571 | L/P |

BL, baseline; *indicates a stop codon.

**Supplementary Table 5. Prevalence of TAB and ZAB Signature Residues in Clonal Baseline ENV Sequences from Participant 1**

|  | **TAB** | | | | | | **ZAB** | | | | | |
| --- | --- | --- | --- | --- | --- | --- | --- | --- | --- | --- | --- | --- |
| **Clone** | **E102** | **I108** | **I201** | **A281** | **Y318** | **F353** | **T63** | **L179** | **T320** | **D325** | **H330** | **332 Glycan** |
| 1 | E | I | I | A | Y | F | T | L | A | D | H | NLS |
| 2 | E | I | I | A | Y | F | T | L | A | D | H | NLS |
| 3 | E | I | I | A | Y | F | T | L | A | D | H | NLS |
| 4 | E | I | I | A | Y | F | T | L | A | D | H | NLS |
| 5 | E | I | I | A | Y | F | T | L | A | D | H | NLS |
| 6 | E | I | I | A | Y | F | T | L | A | D | H | NLS |
| 7 | E | I | I | V | Y | F | T | L | A | D | H | NLS |
| 8 | E | I | I | A | Y | F | T | L | A | D | H | NLS |
| 9 | E | I | I | A | Y | F | T | L | A | D | H | NLS |
| 10 | E | I | I | A | Y | F | T | L | A | D | H | NLS |
| 11 | E | I | I | A | Y | F | T | L | A | D | H | NLS |
| 12 | E | I | I | A | Y | F | T | L | A | D | H | NLS |
| 13 | E | I | I | A | Y | F | T | L | A | D | H | NLS |
| 14 | E | I | I | A | Y | F | T | L | A | D | H | NLS |
| 15 | E | I | I | A | Y | F | T | L | A | D | H | NLS |
| 16 | E | I | I | A | Y | F | T | L | A | D | H | NLS |
| 17 | E | I | I | A | Y | F | T | L | A | D | H | NLS |
| 18 | E | I | I | A | Y | F | T | L | A | D | H | NLS |
| 19 | E | I | I | A | Y | F | T | L | A | D | H | NLS |
| 20 | E | I | I | A | Y | F | T | L | A | D | H | NLS |
| 21 | E | I | I | A | Y | F | T | L | A | D | H | NLS |
| 22 | E | I | I | A | Y | F | T | L | A | N | H | NLS |
| 23 | E | I | I | A | Y | F | T | L | A | D | H | NLS |
| 24 | E | I | I | A | Y | F | T | L | A | D | H | NLS |

ENV, envelope; TAB, teropavimab; ZAB, zinlirvimab.

**Supplementary Figure 1: Phase 1b Study Design and Efficacy Outcomes**


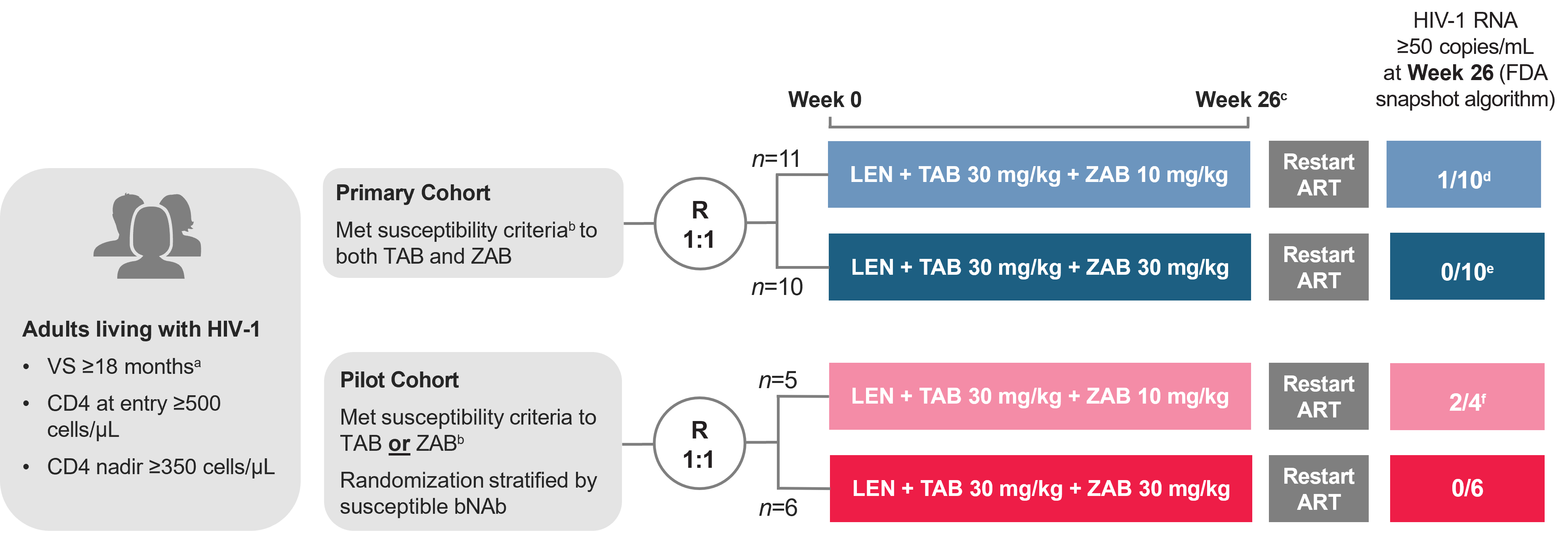


^a^Previous VF was allowed if participants were VS (HIV-1 RNA <50 copies/mL) for ≥18 months prior to screening.

^b^bNAb susceptibility defined as an IC_90_ ≤2 μg/mL by PhenoSense^®^ mAb Assay (Monogram Biosciences).

^c^At Week 26, all participants restarted their previous ART regimen and follow-up continued to Week 52.

^d^One participant withdrew prior to receiving complete study regimen and was excluded from the efficacy analysis.

^e^One participant withdrew for reasons other than adverse event/death or lack of efficacy at Week 12, with last on study HIV-1 RNA <50 c/mL before restarting oral ART.

^f^One participant restarted ART prior to Week 26 due to protocol violation (chronic hepatitis B virus) and was excluded from efficacy analyses.

ART, antiretroviral therapy; bNAb, broadly neutralizing antibodies; c/mL, copies/mL; FDA, Food & Drug Administration; IC_90_, 90% inhibitory concentration; LEN, lenacapavir; mAb, monoclonal antibody; R, randomization; TAB, teropavimab; VF, virologic failure; VS, virologic suppression; ZAB, zinlirvimab.

**Supplementary Figure 2. ENV Baseline and Post-Baseline Amino Acid Sequence Alignment for Each Participant**

**Participant 1:**

**
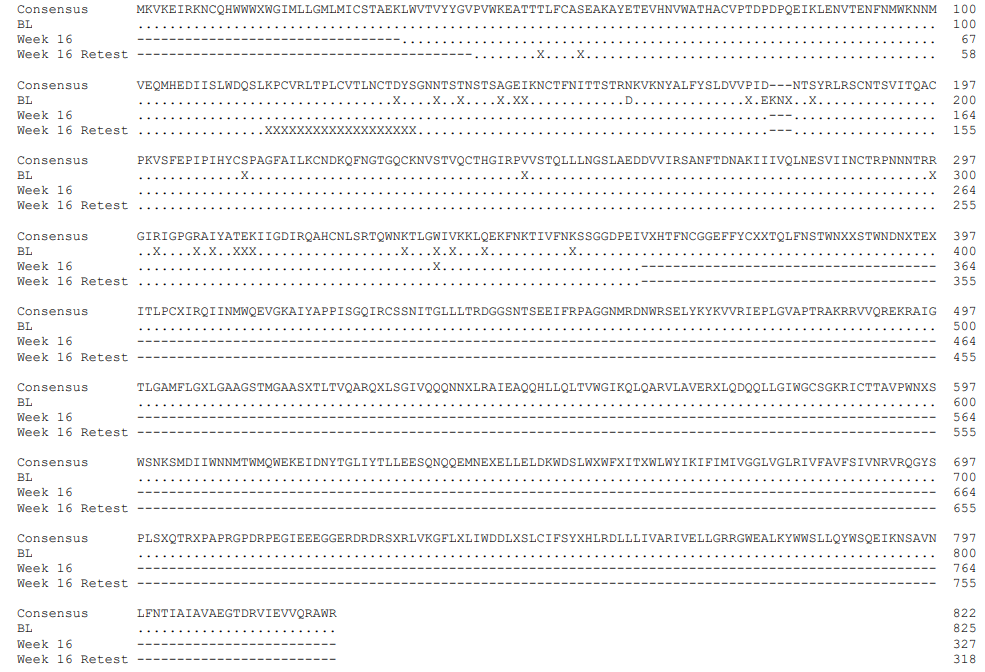
**

**Participant 2:**

**
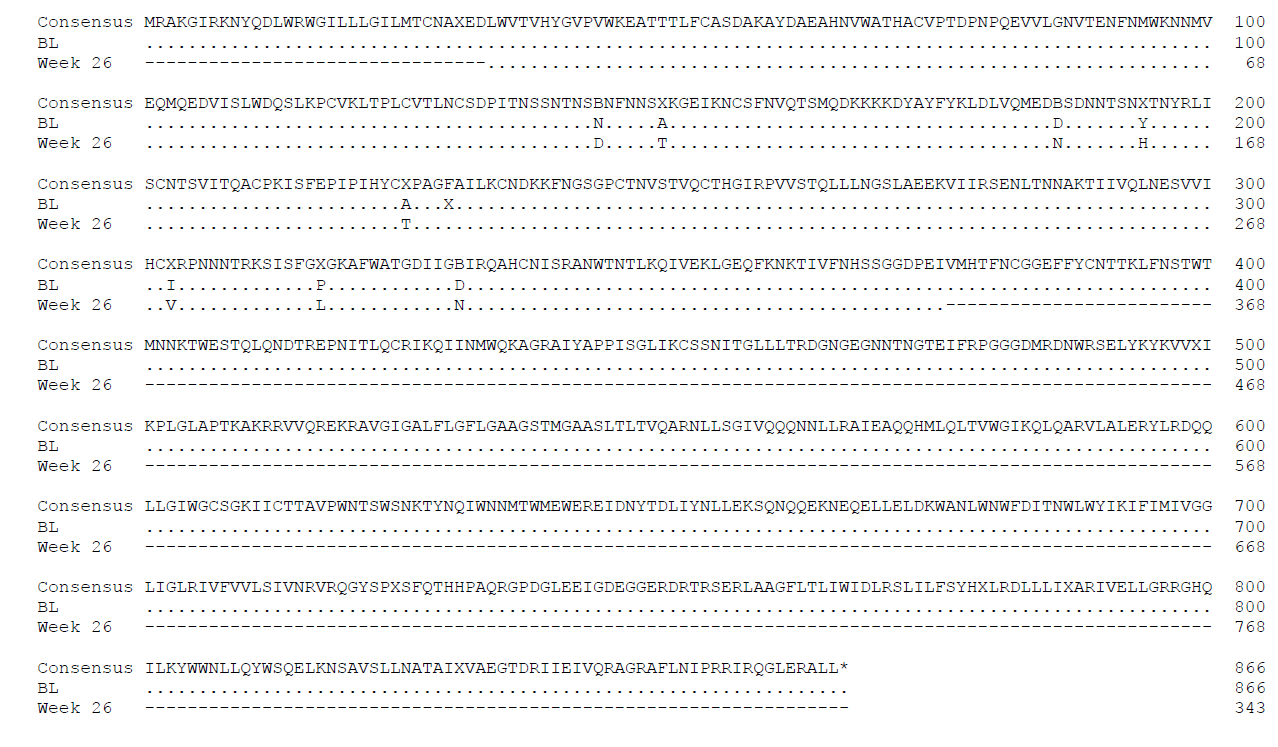
**

**
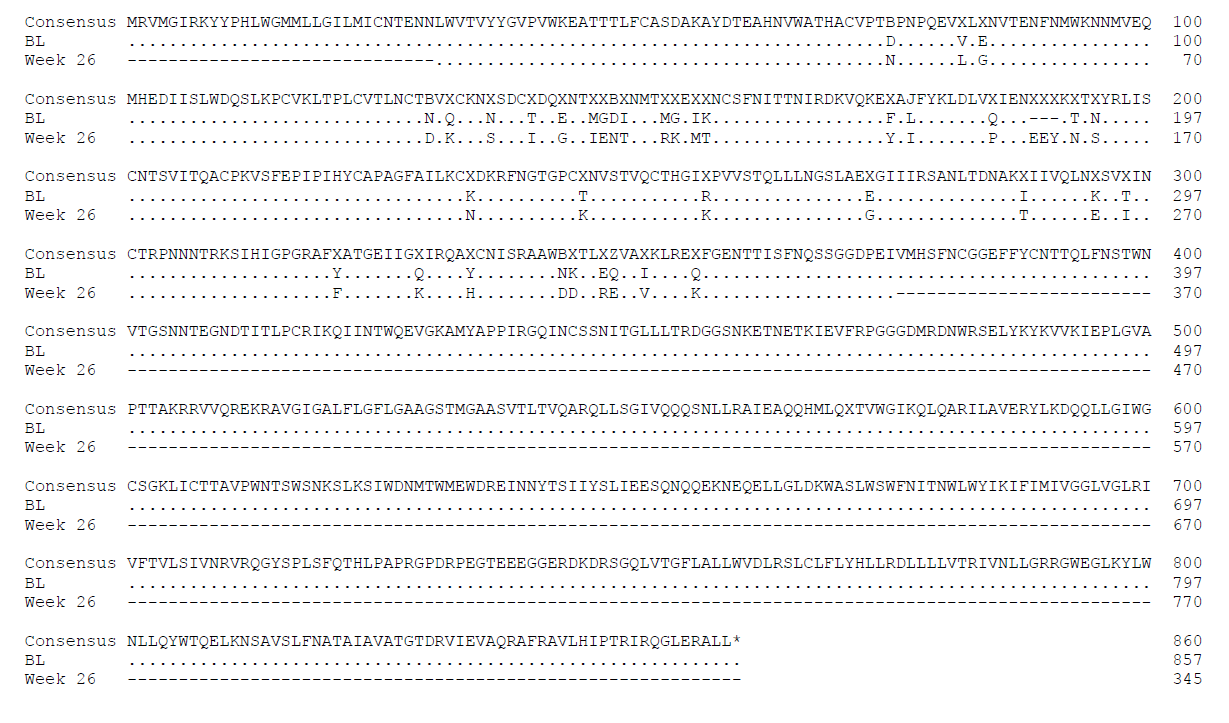
Participant 3:**

Variants are with a prevalence >15% are displayed. Ambiguous amino acids are denoted by “X”. Stretch of "X" from position 117-135 in Participant 1 Week 16 retest sequence denotes insufficient coverage at these positions. A description of each ambiguous amino acid is included in Supplementary Table 4.

ENV, envelope; BL, baseline.

**Supplementary Figure 3. Phylogenetic Analysis of Clonal Baseline and the Week 16 and Week 16 Retest ENV Sequences from Participant 1.**

**A)**


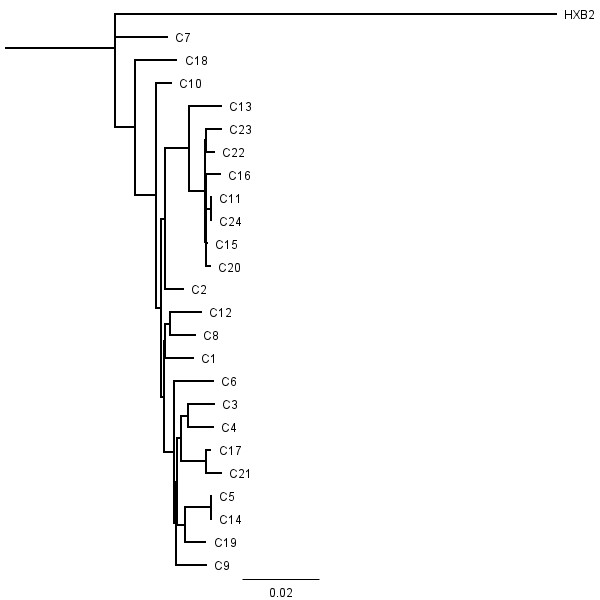


**B)**


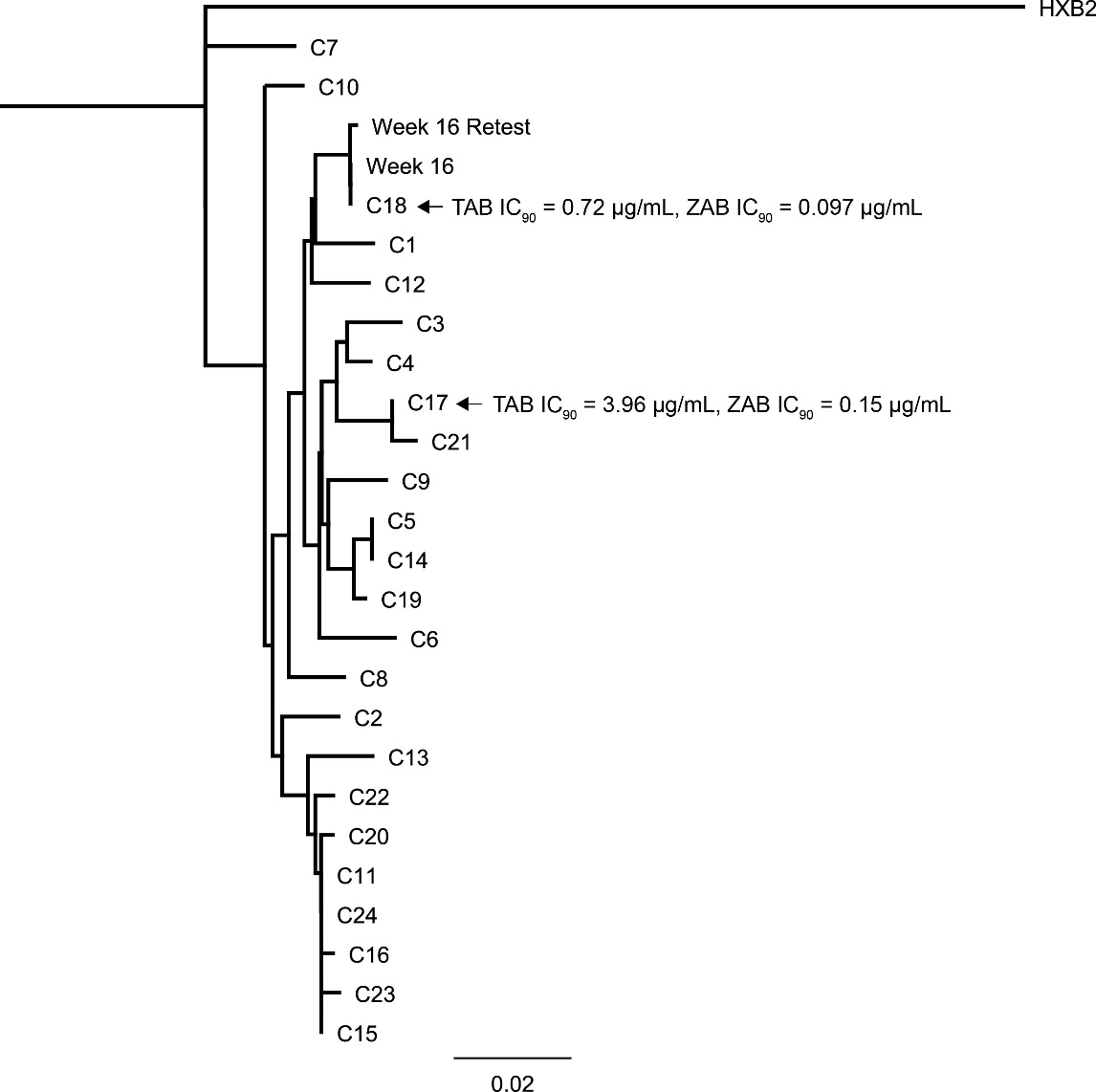


Phylogenetic tree of **A)** full-length ENV (HXB2 gp160 nucleotides 1-2,523) for clones C1-C24 and **B)** the 994 bp stretch of ENV (HXB2 gp120 nucleotides 100–1093) for clones C1-C24 and the consensus ENV sequence obtained at the Week 16 rebound visit. The phylogenetic tree was constructed using Geneious Prime version 2024.0.3, employing the neighbor-joining method with the HKY substitution model.
